# A Biocompatible, Stimuli-Responsive, and Injectable Hydrogel with Triple Dynamic Bonds

**DOI:** 10.3390/molecules25133050

**Published:** 2020-07-03

**Authors:** Yujie Chen, Runjing Zhang, Baiqin Zheng, Chao Cai, Zhen Chen, Hua Li, Hezhou Liu

**Affiliations:** 1 State Key Lab of Metal Matrix Composites, Shanghai Jiao Tong University, Shanghai 200240, China; renee9514zhang@sjtu.edu.cn (R.Z.); cai_chao0102@sjtu.edu.cn (C.C.); sjtu-chenzhen@sjtu.edu.cn (Z.C.); lih@sjtu.edu.cn (H.L.); 2 School of Pharmaceutical and Material Engineering, Jinhua Polytechnic, Jinhua 321017, Zhejiang Province, China; 20050651@jhc.edu.cn

**Keywords:** injectable hydrogel, triple dynamic bonds, natural polysaccharide derivatives, biocompatible, stimuli-responsive

## Abstract

Injectable hydrogels have attracted growing interests as promising biomaterials for clinical applications, due to their minimum invasive implanting approach and easy-handling performance. Nevertheless, natural biomaterials-based injectable hydrogels with desirable nontoxicity are suffering from limited functions, failing to fulfill the requirements of clinical biomaterials. The development of novel injectable biomaterials with a combination of biocompatibility and adequate functional properties is a growing urgency toward biomedical applications. In this contribution, we report a simple and effective approach to fabricate multi-functional CMC-OSA-DTP hydrogels. Two kinds of natural polysaccharide derived polymers, carboxymethyl chitosan (CMC) and oxidized alginate (OSA) along with 3,3′-dithiopropionic acid dihydrazide (DTP) were utilized to introduce three dynamic covalent bonds. Owing to the existence of triple dynamic bonds, this unique CMC-OSA-DTP hydrogel possessed smart redox and pH stimuli-responsive property, injectability as well as self-healing ability. In addition, the CCK-8 and live/dead assays demonstrated satisfying cytocompatibility of the CMC-OSA-DTP hydrogel in vitro. Based on its attractive properties, this easy-fabricated and multi-functional hydrogel demonstrated the great potential as an injectable biomaterial in a variety of biomedical applications.

## 1. Introduction

Hydrogels bear a resemblance to extra cellular matrix (ECM) and enable cells to adhere, proliferate and differentiate, resulting from the three-dimensional crosslinked hydrophilic networks, porous framework and high water content [1,2]. Therefore, hydrogels have stood out among other candidates as promising biomaterials, in particular for tissue engineering [3], controlled drug release [4], and wound healing [5].

With considerable advances in biomaterials for clinical applications, functional properties of hydrogels are gaining importance to meet practical requirements. Currently, open surgical procedures to implant pre-formed hydrogels are confronted with increasing infection risks, overlong recovery time, and excessive healthcare expenses [6]. Injectable hydrogels provide a favorable solution to overcome these concerns as they can accommodate irregular defect margin filling [7], and reach wounds in very deep tissues [8] with minimum invasiveness and ease of handling [9]. Relevant reported studies on injectable hydrogels for biomedical applications have achieved excellent operability and biocompatibility [10]. In addition, “smart” hydrogels, which can respond to various stimuli, such as light [11], heat [12], pH [13], and redox environment [14], have been intensively explored because of their great potential in biomedical applications, including tissue engineering, biosensors, drug delivery systems, and artificial muscle [15,16]. For example, drugs can be released under control responding to tumor microenvironment with acidosis and reducing agents, such as glutathione [17], which could be utilized to develop targeted drug delivery systems. Meanwhile, the responsive sol-gel transition facilitates rapid and efficient removal from the body [18].

Among various injectable hydrogels in medical implanting fields, natural polysaccharide-based injectable hydrogels are especially attractive. Alginate, extracted from brown seaweeds, is a typical natural polysaccharide-based polymer with remarkable biocompatibility [19] and able to form hydrogels through ionic crosslinking with Ca^2+^ [20]. Due to the slow and poorly controlled degradation of alginate, oxidized alginate with better degradation kinetics [21] and more reactive groups [22] has been widely used in biocompatible and injectable hydrogel fabrications. Chitosan, a linear polysaccharide partially deacetylated from chitin [23], has also aroused much attention in injectable biomaterials. Taking advantage of the innate antibacterial property of chitosan, Li et al. developed an injectable chitosan-based hydrogel with antimicrobial and antifouling properties for bioengineering applications [24]. Furthermore, to solve the water-insolubility problem of chitosan, various water-soluble chitosan derivatives were successfully developed toward medical applications [17,25]. Attributed to their desirable intrinsic properties, such as nontoxic, biocompatibility, low immune rejection, and excellent renewability, natural polysaccharide-based injectable hydrogels have showed promising potentials in biomedical fields. Nevertheless, their clinical applications are suffering from limited functions, such as the lack of mechanical strength [7], or environmental-responsive capability [26], failing to fulfill the requirements as effective implanting materials. Therefore, the development of novel injectable biomaterials with a combination of multiple functional properties without sacrificing biocompatibility is in high demand.

Herein, we proposed a facile methodology to fabricate high-performance hydrogels-employed biocompatible polysaccharide derived polymers, carboxymethyl chitosan (CMC), and oxidized alginate (OSA), along with 3,3′-Dithiopropionic acid dihydrazide (DTP). The CMC-OSA-DTP hydrogel with the presence of triple dynamic bonds was successfully fabricated. Compared with the single bond strategy, integrating different dynamic chemistries could impart more functions to hydrogels [27]. Double dynamic bonds strategy is commonly used in self-healing hydrogels [28,29]. However, for all we know, there is a lack of simple approaches to introduce triple dynamic bonds in one injectable hydrogel network. In our work, the synergy of three diverse dynamic bonds allowed the combination of multiple satisfying properties. Utilizing dynamic Schiff bases imine bonds and acylhydrazone bonds to form double cross-linking networks achieved better mechanical properties. Meanwhile, the coexistence of acylhydrazone bonds and disulfide bonds endowed hydrogels with dual environmental-responsiveness. Additionally, CMC-OSA-DTP hydrogels also possessed excellent injectable and self-healing ability owing to the debonding-rebonding behaviors of dynamic bonds. Furthermore, due to the gentle properties of safe ingredients, the resulting hydrogel also exhibited remarkable nontoxicity and cytocompatibility as demonstrated by CCK-8 and live/dead assays using MG-63 cells. We envision this smart injectable hydrogel based on triple dynamic bonds could offer an opportunity to develop ideal candidates as injectable implanting biomaterials and broaden the application environments in biomedical fields. In general, a successful cell encapsulation and delivery system requires hydrogels with excellent biocompatibility and appropriate mechanical properties for cell immobilization and deposition. Furthermore, due to the redox and pH responsiveness, CMC-OSA-DTP hydrogels would be very useful in drugs and bioactive molecules delivery. Both acidic and reducing conditions could trigger the fast release of drug from the CMC-OSA-DTP hydrogel, which means the hydrogel could release drug efficiently in targeted environment, like the stomach and the tumors, and minimalize the amount of drug release in normal physiological environment. Based on these attractive properties, CEC-OSA-DTP hydrogels demonstrate great potential in a variety of biomedical applications.

## 2. Results and Discussion

### 2.1. Design and Construction of CMC-OSA-DTP Hydrogels

**Figure 1:**
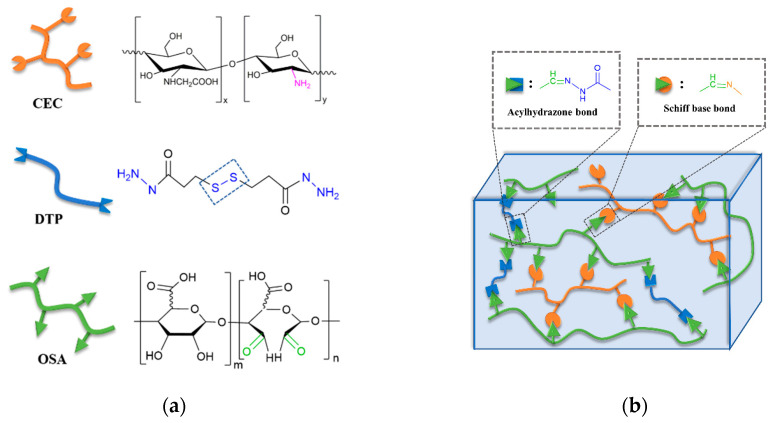
Synthesis of carboxymethyl chitosan (CMC)-oxidized alginate (OSA)-dithiopropionic acid dihydrazide (DTP) hydrogels. (**a**) Chemical illustrations and structures of CMC, DTP, and OSA; (**b**) Schematic illustration of the hydrogel cross-linking strategy.

As depicted in Figure 1, two kinds of water-soluble naturally derived polymers, carboxymethyl chitosan (CMC) and oxidized alginate (OSA), were chosen to fabricate injectable hydrogels. The oxidized alginate (OSA) with aldehyde groups was obtained via the periodate oxidation reaction, modified on our previous work [30]. The oxidation reaction was confirmed by ^1^H-NMR spectrum of SA and OSA (Figure S1). Compared with sodium alginate (SA), the appearance of two new OSA signals at 5.15 and 5.37 ppm correspond to the hemiacetalic proton formed between aldehyde and neighboring hydroxyl groups [31]. The average oxidation degree was 84.2% (Table S1). 3,3′-Dithiopropionic acid dihydrazide (DTP) that contains disulfide bond and hydrazide groups was also employed in this hydrogel. DTP was synthesized according to the literature [32]. The chemical structure was characterized by ^1^H-NMR spectroscopy (Figure S2) and the experimental values were in good agreement with the theoretical values.

The backbone network of the CMC-OSA-DTP hydrogel is illustrated in Figure 1b. The CMC-OSA-DTP hydrogel network was dual crosslinked by dynamic Schiff bases imine bonds and acylhydrazone bonds. OSA functionalized with aldehyde groups cross-linked CMC with amine to form imine bonds via Schiff base reaction. Schiff base linkages are able to form under mild environments with a high reaction rate, which are ideal to prepare biologically relevant hydrogels [33]. Besides, dynamic acylhydrazone covalent bonds were also obtained by the reaction between aldehyde groups of OSA and hydrazide groups of DTP. The appropriate the ratio of three components in the dual cross-linked hydrogel is OSA: DTP + CMC = 1:1. The total concentration of all ingredients was fixed at 8.25 wt%. FT-IR analysis (Figure S3) confirmed the cross-linking strategy. A new peak at 1665 cm^−1^ appeared in the FT-IR spectra of CMC-OSA-DTP hydrogel, corresponding to the C=N stretching vibrations in imine and acylhydrazone bonds. In order to distinguish peaks of acylhydrazone bonds and imine bonds at similar wavenumbers and verify the formation of different bonds, OSA-DTP and CMC-OSA hydrogels were also prepared. The absorption peak of OSA-DTP hydrogel at around 1664 cm^−1^ in the FT-IR spectra, were attributed to the formation of acylhydrazone bonds [34,35]. Meanwhile, the FT-IR spectra of CMC-OSA hydrogel exhibited the characteristic absorption peak at 1666 cm^−1^ of stretching vibration of imine bonds, verifying the Schiff bases reactions [36].

### 2.2. Dual Stimili-Responsiveness of CMC-OSA-DTP Hydrogels

Environmental responsiveness is the most typical property of hydrogels with dynamic bonds. Dynamic disulfide bonds and acylhydrazone bonds contained in CMC-OSA-DTP hydrogels endowed hydrogels with dual responsive capability to redox environments and pH changes by sol-gel transition behaviors. Disulfide bonds, which play a vital role in protein folding and assembly, could break in reducing environments and reform in oxidizing environments via thiolate/disulfide exchange reactions [35]. As for acidic environments, the CMC-OSA-DTP hydrogel underwent acylhydrazone exchange in response [37]. Both disulfide and acylhydrazone bonds are compatible with physiological environments, thus they are suitable for self-healing hydrogels in biomedical applications, especially for controlled drug release.

**Figure 2:**
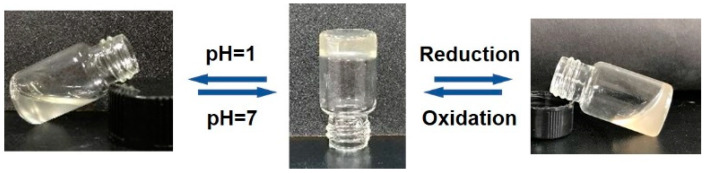
Digital photos demonstration of environmental responsive sol-gel transition of CMC-OSA-DTP hydrogels.

As illustrated in Figure 2, the CMC-OSA-DTP hydrogel turned into colloidal sol state by introducing the reductant 1,4-dithiothreitol (DTT) as the initially formed disulfide bonds were reduced to the free thiol groups. Nevertheless, after the addition of oxidant H_2_O_2_, the solution reformed into hydrogel under oxidizing environments. Besides redox responsiveness, the hydrogel also displayed reversible sol-gel transitions as pH changed. The hydrogel was decomposed into liquid with the addition of HCl and recovered hydrogel state in a neutral environment by adding triethylamine (TEA). It is the acylhydrazone dynamic bonds that broke at low pH and reformed at high pH, which explained the pH responding sol-gel transitions of CMC-OSA-DTP hydrogels.

### 2.3. Self-Healing Ability of CMC-OSA-DTP Hydrogels

**Figure 3:**
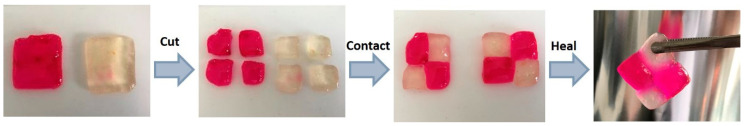
Digital photos of the self-healing process of CMC-OSA-DTP hydrogel pieces (red pieces were stained with rhodamine B for visual discrimination.).

Another key characteristic of hydrogels based on dynamic covalent bonds is their self-healing ability. The self-healing performance of CMC-OSA-DTP hydrogels is demonstrated in Figure 3. Each square hydrogel (one was dyed red using rhodamine B for easier visualization) was cut into four pieces. Smaller hydrogel pieces of the same color were placed diagonally. When four smaller hydrogel pieces were brought into contact, a new square hydrogel was obtained after 10 min. The adhesion strength after self-healing was large enough to support its own weight. The excellent self-healing action was triggered by multiple dynamic bonds between functional groups, which accomplished re-bonding after breakage [37].

**Figure 4:**
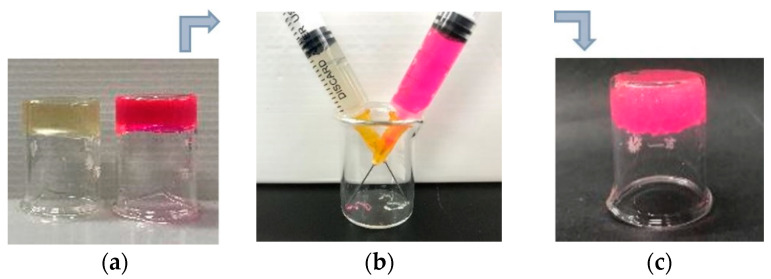
Digital photos of injectable CMC-OSA-DTP hydrogels before and after injecting. (**a**) The preparation of two different hydrogels. The red hydrogel was stained with rhodamine B for visual discrimination; (**b**) hydrogels passed through needles and injected in the same beaker. (**c**) The integral hydrogel formed after self-healing.

As for injectable materials, the self-healing ability is much more critical because they need to bear high strains and network rupture through the syringe before reforming bulk hydrogels. As shown in Figure 4, the resultant CMC-OSA-DTP hydrogel was able to be extruded through a 25-gauge needle without clogging. After injection, the two hydrogels with different colors (one was stained by rhodamine B for visual discrimination) could self-heal and form a new integral hydrogel, resulting from the dynamic uncoupling and recoupling ability of diverse covalent bond linkages in the CMC-OSA-DTP hydrogel.

**Figure 5:**
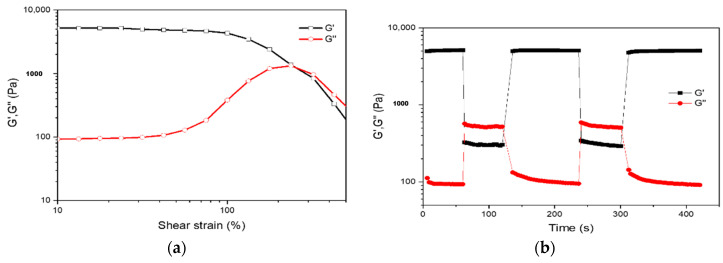
(**a**) Strain amplitude sweep measurements from 10% to 500% strain of CMC-OSA-DTP hydrogels. (**b**) Continuous step-strain measurements at alternate 0.1% and 500% strain of CMC-OSA-DTP hydrogels.

Another significant and readily measurable method to represent the self-healing ability is the dynamic rheology test [2]. The amplitude sweep measurement was performed firstly in order to ascertain the linear viscoelastic region of the hydrogel and find out the critical strain value required to break the hydrogel network. As demonstrated in Figure 5a, with increasing strain, the storage moduli (G′) dropped while the loss moduli (G″) raised. The storage and loss moduli curves displayed a crossover at the strain of 240%, indicating the gel network disruption. Thus, the critical strain point of hydrogel to convert into the sol state was found here to be γ = 240%. Accordingly, alternate step-strain experiments were performed to determine self-recovery properties of the hydrogel following network disruption. As presented in Figure 5b, the CMC-OSA-DTP hydrogel was subjected to a high magnitude strain (γ = 500%) with evident inversion of the storage (G′) and loss moduli (G″), indicating hydrogel network fracture. When the high strain was retreated and a low strain (γ = 0.1%) was applied, the hydrogel displayed nearly full mechanical property recovery within a few seconds after severe strain deformation. The self-healable performances are reproducible upon additional stress-relaxation cycles of breaking and reforming, highlighting the self-healing ability of hydrogels due to the reversible exchange and reformation of dynamic bonds.

### 2.4. Mechanical Properties and Morphology Observations of Hydrogels

**Figure 6:**
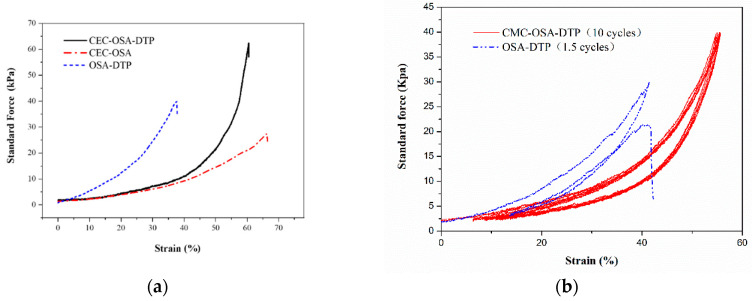
(**a**) Typical compressive stress-strain curves of hydrogels with different compositions. (**b**) Successive loading-unloading compressive tests of CMC-OSA-DTP hydrogel (10 cycles) and OSA-DTP hydrogel (1.5 cycles).

The combination of dynamic bonds not only endows the hydrogel with environmental responses, but also increases the mechanical toughness. The double covalent cross-linking network structure of CMC-OSA-DTP hydrogel was constructed by dynamic Schiff bases imine and acylhydrazone bonds. CMC-OSA-DTP hydrogel exhibited excellent compressive strength because of the synergistic effect of dynamic bonds. The compressibility of the hydrogel based on double cross-linking networks (CMC-OSA-DTP) and hydrogels based on single cross-linking network (CMC-OSA and OSA-DTP) was measured to evaluate the mechanical strength. The compression performances of the resultant hydrogels are presented in Figure 6.

**Figure 7:**
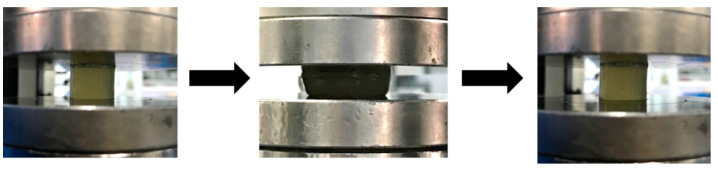
Digital photos of CMC-OSA-DTP hydrogels before and after compressive loading.

Remarkably, obvious distinctions on the mechanical properties of hydrogels with different compositions were noticed. As shown in Figure 6a, CMC-OSA-DTP hydrogel exhibited the best mechanical load bearing capacity among the three samples, reaching around 65 kPa. Nevertheless, the compressive strength of CMC-OSA hydrogel possessing higher elastic moduli was nearly 25 kPa, less than half of CMC-OSA-DTP hydrogel. Similarly, OSA-DTP hydrogel also had mediocre compression bearing capacity (40 kPa). Cyclic tests highlighted the mechanical differences between the hydrogels based on double cross-linking networks and single network. As shown in Figure 6b, the mechanical hysteresis loops of CMC-OSA-DTP hydrogel were similar in successive loading-unloading compression cyclic tests, demonstrating remarkable recovery of mechanical property of hydrogels adopted double cross-linking networks strategy. In contrast, OSA-DTP hydrogel failed to regain its original compressive strength from the second cycle and the mechanical strength of CMC-OSA was too weak to perform the compression cycles for comparison. Digital photos of CMC-OSA-DTP hydrogels during loading-unloading compression tests are shown in Figure 7. Mechanical tests indicated that the mechanical reinforcement of hydrogels was significantly improved by the presence of double cross-linking networks. The combination of dynamic Schiff bases imine and acylhydrazone bonds provide polymer chain entanglement and stress dissipation mechanisms [38], which contributed to reaching an improved balance between strength and elasticity.

**Figure 8:**
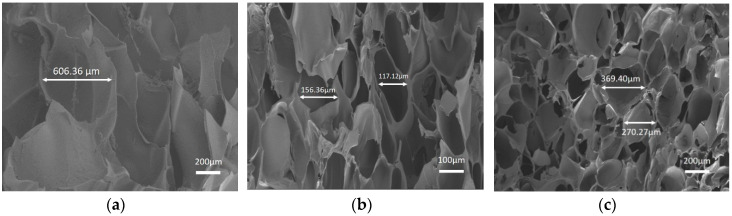
SEM images of freeze-dried hydrogels with different compositions. (**a**) OSA-DTP hydrogel; (**b**) CMC-OSA-DTP hydrogel; (**c**) CMC-OSA hydrogel.

To further explain the strength differences among the fabricated hydrogels above, the architecture of hydrogels, which makes a dominant contribution to scaffolds mechanical performance, was studied by scanning electron microscope (SEM) images in order to show the cross-sectional morphology. As presented in Figure 8, after lyophilization, representative porous interconnected microstructures were observed in all CMC-OSA-DTP, CMC-OSA, and OSA-DTP hydrogel scaffolds appeared controlled. With different ingredients ratio, hydrogel scaffolds with different pore sizes were observed. It was clear that hydrogels with the coexistence of CMC&DTP ingredients (Figure 8b) displayed smaller pore sizes, ranging from 120 μm to 180 μm. However, the honeycomb-like ordered structures of CMC-OSA and OSA-DTP hydrogels displayed larger pore sizes in the scaffolds (Figure 8a,c). The various pore sizes among different hydrogels might be explained that scaffolds with both CMC and DTP ingredients were prone to generate small sheet linkages and form small pore sizes because of double cross-linking networks containing more linking points. It is common knowledge that the mechanical performance of porous materials depends largely on pore structures. The compact structure with smaller pores can make a significant contribution to enhancing the compressive modulus. It is reported that the mechanical strength of porous scaffolds would make remarkable improvements by forming chemical bonds through the cross-linking reaction between chemical functional groups [39]. Compared with other hydrogels based on single cross-linking network (CMC-OSA, OSA-DTP) in our study, the CMC-OSA-DTP hydrogel possesses smaller pore sizes due to their increased cross-linking points. As a result, the improvement of compressive strength of CMC-OSA-DTP was achieved.

### 2.5. Cytocompatibility and Cell Culture of Hydrogels

In order to apply this multi-functional CMC-OSA-DTP injectable hydrogel in clinical therapy, sufficient biocompatibility requirements should be met primarily. To study the biological performance of resultant hydrogels for future applications, we have used human osteoblast-like cells, MG-63 cells, which have been widely employed to prove the biocompatibility of materials for biomedical applications [40,41].

**Figure 9:**
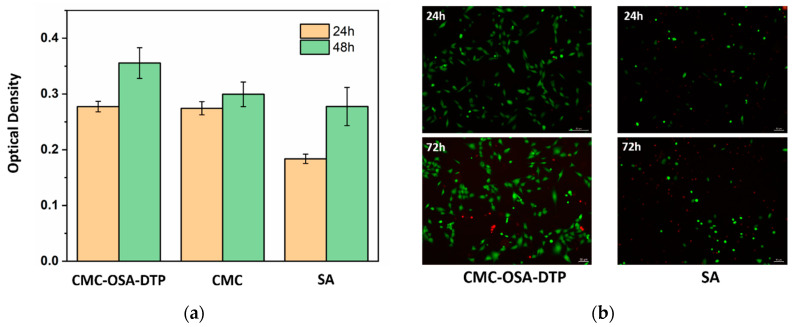
Cytocompatibility evaluation of hydrogels. (**a**) MG-63 cell proliferation measured 24 and 48 h after seeding on CMC-OSA-DTP hydrogel, pure alginate hydrogel (SA), and pure chitosan sticky liquid (CMC). (**b**) LIVE/DEAD assay images of MG-63 cells encapsulated within CMC-OSA-DTP hydrogel and pure alginate hydrogel (SA) for 24 h, 72 h.

The ability of the hydrogels to interact with cells was evaluated by seeding MG-63 cells on the surface of CMC-OSA-DTP hydrogels to approve the cytocompatibility. Naturally occurring derivatives, alginate (SA) and carboxymethyl chitosan (CMC), were chosen as control materials due to their well-known biocompatibility. After seeding the cells, adhered and proliferated cells were measured at 24 and 48 h by the CCK-8 assay. As shown in Figure 9a, during the incubation period, the MG-63 cells seeded on the CMC-OSA-DTP hydrogel exhibited a higher proliferation rate than on the pure alginate hydrogel in 24 h. At 48 h, CMC-OSA-DTP hydrogel displayed the best performance to facilitate cell proliferation among all samples, suggesting excellent cytocompatibility of CMC-OSA-DTP hydrogels comparable to natural polysaccharide derived biomaterials.

To further assess the potential of the CMC-OSA-DTP hydrogel in cell culture, the 3D MG-63 cell viability within the hydrogels in vitro was determined by a live-dead cell staining kit at different time intervals. After incubation for one day and three days, the cells were stained and imaged by fluorescence microscope. Figure 9b showed that there was a higher ratio of green/red dots in CMC-OSA-DTP hydrogel compared with the pure alginate hydrogel, demonstrating higher viability of MG-63 cells within CMC-OSA-DTP hydrogel [42]. Besides the mild fabricating environments of the hydrogel, the outstanding cytocompatibility is also related to the biocompatibility of naturally derived backbone materials and the porous structures suitable for cell growth [39].

Above all, in cell assays of CMC-OSA-DTP hydrogel, MG-63 cells displayed proliferative capacity and high viability. Besides the long-term stability of hydrogels was checked. OSA-CEC-DTP hydrogels were immersed in PBS solution (pH = 7.4) to intimate the environment of human body. After two weeks, the hydrogel was firstly washed to remove excess degraded polysaccharides and salts before freeze-drying, then the hydrogel samples were weighted after freeze drying. The average weight of dried samples is very close to the weight of hydrogel ingredients. Therefore, according to these results, the loss of hydrogel can be nearly ignored in PBS environment. The stability of hydrogels in vitro is basically confirmed. These biological characteristics make CMC-OSA-DTP hydrogel a promising candidate as implantation biomaterials in biomedical applications.

## 3. Materials and Methods

### 3.1. Materials

Sodium alginate (SA, 99.5%, 200–500 mPa·s), sodium periodate (AR, 99.5%), ethylene glycol (AR, 99%), carboxymethyl chitosan (CMC, BR), dimethyl 3,3′-dithiopropionate (99.5%), triethylamine (99.5%) and hydrazine hydrate (98.0%) were purchased from Shanghai Macklin Biochemical Co., Ltd. (Shanghai, China). Methanol (99.9%) was purchased from J&K Scientific Ltd. (Shanghai, China). 1,4-Dithiothreitol (DTT, 99%) was purchased from Aladdin Chemical Co. Rhodamine B was purchased from Chemiejoy Biotechnology Co., Ltd. (Shanghai, China). Phosphate buffered saline (PBS, pH = 7.4) was purchased from BBI Life Sciences Corporation (Shanghai, China). MG-63 cell lines were purchased from the Type Culture Collection of the Chinese Academy of Sciences (Shanghai, China). Cell counting kit-8 was purchased from Dojindo Molecular Technologies Inc. (Tokyo, Japan). LIVE/DEAD™ Viability/Cytotoxicity Kit (L3224) was purchased from Thermo Fisher Scientific (Waltham, MA, USA).

### 3.2. Synthesis of Oxidation Sodium Alginate

The oxidized sodium alginate (OSA) with aldehyde groups was obtained via periodate oxidation reaction, modified on our previous work [43]. In brief, 10 g sodium alginate (SA) and 11 g sodium periodate were sequentially dissolved in 50 mL of ethanol and 50 mL of DI water, respectively, under continuous stirring in black flasks. After mixing the two dispersions, the mixture kept in the dark was stirred magnetically for 6 h. Then, the oxidation reaction was quenched by introducing 15 mL of ethylene glycol followed by another 2 h of stirring. Thereafter, the reaction mixture was poured into 1.0 L of ethanol containing 3.0 g NaCl to purify the oxidized alginate by precipitation. After re-precipitation by suction filtration, the polymer was collected. Continually, the crude polymer was dialyzed against distilled water for 3 days with several changes of water every day. Finally, OSA was regained by freeze-drying method.

### 3.3. Synthesis of 3,3′-Dithiopropionic acid dihydrazide (DTP)

3,3′-Dithiopropionic acid dihydrazide (DTP) was synthesized according to the literature [32]. Briefly, 2.04 g dimethyl 3,3′-dithiopropionate (1.22 g/cm^−3^, 8.3 mmol) was dissolved in 50 mL MeOH with the addition of 2.55 mL hydrazine hydrate (50 mmol) and the mixture stirred for 12 h at room temperature. DTP was obtained by filtering and washed with MeOH and water.

### 3.4. Fabrication of CMC-OSA-DTP, CMC-OSA, OSA-DTP Hydrogels.

The in situ formation of CMC-OSA-DTP hydrogel was fabricated through homogeneously mixing OSA with water-soluble CMC and DTP at room temperature. To imitate the body environment, phosphate buffer saline (PBS, pH 7.4) was prepared as the solvent. 0.04 g DTP and 0.14 g CMC were dissolved in 2 mL PBS. The homogeneous CMC&DTP/PBS solution was mixed with the PBS solution of OSA (0.1 g/mL) under magnetically stirring. After stirring the reaction mixture by vortex for a few seconds, homogeneous hydrogel formation can be recognized by the cessation of flow on inclining the flask.

The fabrication methods of CMC-OSA hydrogels and OSA-DTP hydrogels were similar to that of CMC-OSA-DTP hydrogels. The total concentration of all hydrogels was fixed at 8.25 wt%. To keep same molar ratio of aldehyde groups in OSA to amino groups in CMC or hydrazide groups in DTP, the ratio of CMC:OSA = 1:1 in CMC-OSA hydrogels, while the ratio of DTP:OSA = 1:3.

### 3.5. Characterization

#### 3.5.1. Dual Stimuli-Responsive Tests of Hydrogels

To verify the redox responsiveness of CMC-OSA-DTP hydrogels, 10 mg DTT was added as reductant to 1 g hydrogel. After the addition of 8 µL H_2_O_2_, the sol-gel transition could be observed. To observe the pH-triggered gel-sol transition, 1 g CMC-OSA-DTP hydrogel was treated with 5 M HCl solution (50 µL) with vortex overnight. Hydrogel reformed after the addition of 40 µL triethylamine to adjust pH = 7.

#### 3.5.2. Mechanical Tests of Hydrogels

The compressibility of the CMC-OSA-DTP, CMC-OSA, and OSA-DTP hydrogels was measured using a universal testing machine (CMT 5105, SANS Testing Machine Co., LTD, Shen Zhen, China). Each as-prepared hydrogel used for the compression test was with a thickness of 12 mm and a diameter of 12 mm. The crosshead speed was 20 mm min^−1^ at room temperature.

#### 3.5.3. Dynamic Rheological Measurements of Hydrogels

The oscillatory rheological experiments were carried out within the rheometer (Gemini 200 HR, Bohlin Instruments, Worcestershire, UK) equipped with a 25 mm-diameter flat plate and the gap was fixed on 1 mm. The amplitude sweep measurement over strain γ ranging from 10% to 500% was performed at a constant frequency of 1 Hz. The stress-relaxation experiments were conducted switching between shear deformation strain γ = 500% and relaxation strain γ = 1% at the constant frequency of 1 Hz in a stepwise manner at room temperature.

#### 3.5.4. Cytocompatibility Evaluation of Hydrogels In Vitro

MG-63 cell lines were employed to analyze the biocompatibility of materials for future applications [41]. In the CCK-8 assay, CMC-OSA-DTP and SA hydrogels were fabricated in a 96-well culture plate containing PBS solution. All the ingredients were sterilized by UV light for 3 h and fabricated hydrogels were sterilized by ethanol overnight and rinsed with PBS. MG-63 cells were seeded at a density of 8 × 10^3^ cells per well. The adhered cells on different test samples immersed in Dulbecco’s Modified Eagle’s Medium (DMEM) were incubated under a humidified atmosphere with 5% CO_2_ at 37 °C. The cell proliferation was evaluated at 24 h and 48 h. Next, 10 µL cell counting kit-8 (CCK-8) solution was added per well in the dark and the MG-63 cells were incubated for another 2 h. The intensity of MG-63 was measured using a microplate reader (Thermo MULTISKAN MK3, Waltham, MA, US) at a wavelength of 450 nm [44]. As for the 3D cell culture in hydrogels, MG-63 cells were suspended in PBS before hydrogel gelation. The cell/hydrogel construct with a density of 5 × 10^5^ cells mL^−1^ were put into a 12-well culture plate containing 2 mL DMEM per well and then incubated at 37 °C under 5% CO_2_ atmosphere. The viability of MG-63 cells was visualized by LIVE/DEAD™ Viability/Cytotoxicity Kit using an inverted fluorescence microscope (Leica DMi8, Wetzlar, Germany).

## 4. Conclusions

In conclusion, a biocompatible, dual stimuli-responsive and injectable CMC-OSA-DTP hydrogel with triple dynamic covalent bonds was reported. Due to the synergetic effects of diverse dynamic bonds, this injectable CMC-OSA-DTP hydrogel exhibited dual environmental responsiveness, mechanical bearing, and self-healing ability. In addition, based on natural polysaccharide derived polymer ingredients, CMC-OSA-DTP hydrogel also displayed excellent biocompatibility to expand its future applications in tissue engineering and drug delivery. Hopefully, this work may enlighten the design and fabrication of natural polymer to high-performance hydrogels as biomedical materials in the near future.

## Supplementary Materials

Figure S1: ^1^H-NMR spectrum of SA, OSA; Figure S2: ^1^H-NMR spectrum of DTP; Figure S3: FT-IR spectra of oxidized sodium alginate (OSA), OSA-DTP hydrogel, CMC-OSA-DTP hydrogel, CMC-OSA hydrogel and carboxymethyl chitosan (CMC); Figure S4: Swelling ratio of CMC-OSA-DTP hydrogel; Table S1: Degree of aldehyde substitution of OSA. References [45,46] are cited in the supplementary materials.
