# Supplementary material for: A Biocompatible, Stimuli-Responsive, and Injectable Hydrogel with Triple Dynamic Bonds
Source: Molecules. 2020 Jul 3;25(13):3050. doi: 10.3390/molecules25133050 (PMC7412394; doi:10.3390/molecules25133050)
Supplement: Supplementary file 1 [file molecules-25-03050-s001.pdf]

molecules

Supplementary Materials

A Biocompatible, Stimuli-Responsive, and Injectable Hydrogel with Triple Dynamic Bonds

Yujie Chen [1,*,†], Runjing Zhang [1,†], Baiqin Zheng [2], Chao Cai [1], Zhen Chen [1] and Hua Li [1] and Hezhou Liu [1,*]

[1] State Key lab of Metal Matrix composites, Shanghai Jiao Tong University, Shanghai, Shanghai 200240, China; renee9514zhang@sjtu.edu.cn (R.Z.); cai_chao0102@sjtu.edu.cn (C.C.); sjtu-chenzhen@sjtu.edu.cn (Z.C.); lih@sjtu.edu.cn (H.L.);
[2] School of Pharmaceutical and Material Engineering, Jinhua Polytechnic, Jinhua, Zhejiang Province 321017, China; 20050651@jhc.edu.cn
* Correspondence: yujiechen@sjtu.edu.cn, Tel.: +86-21-3420-2549 (Y.C.); hzhliu@sjtu.edu.cn; Tel.: +86-21-3420-2549 (H.L.)
† These authors contributed equally to this work.

Characterization of Oxidation of Sodium Alginate

The oxidation reaction was confirmed by ^{1}H NMR spectrum of SA and OSA, as shown in Figure S1. Compared with sodium alginate (SA), the appearance of two new OSA signals at 5.15 and 5.37 ppm corresponds to the hemiacetalic proton formed between aldehyde and neighboring hydroxyl groups[31].

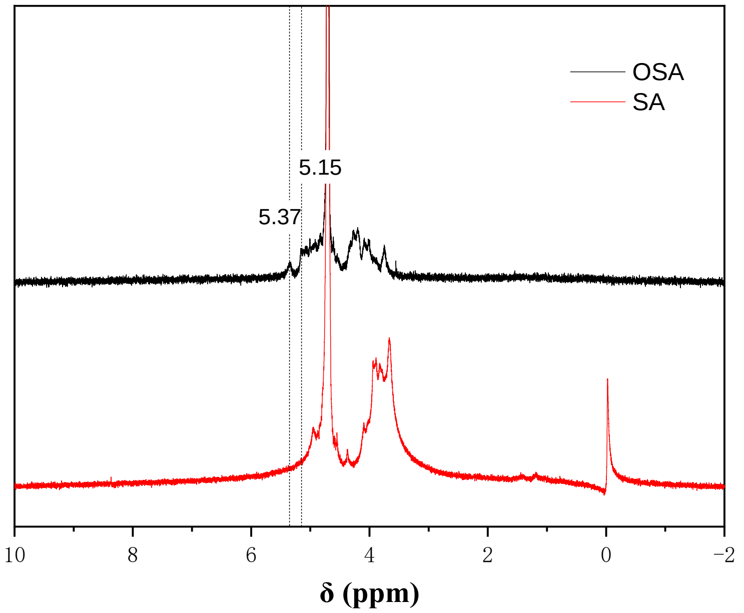

Figure S1. ^{1}H NMR spectrum of SA, OSA.

Determination of Aldehyde Substitution Degree of Oxidized alginate

The degree of aldehyde substitution of OSA was measured by hydroxylamine hydrochloride titration method[45]. 500 mL of 0.25 M hydroxylamine hydrochloride solution was prepared by adding 5.39g dried hydroxylamine hydrochloride and 2ml methyl orange

reagent (0.05 wt%) in DI water. 0.20g OSA was dissolved in 25 ml prepared hydroxylamine hydrochloride solution under magnetically stirring. The concentration of aldehyde groups was determined by titration of released HCl with 0.1 M NaOH solution. The volume of consumed NaOH solution was recorded. The red-to-yellow end point was achieved.

The oxidation degree (OD) was calculated according to following formula:

$$\Delta V_{NaOH} \times 0.001 \times c_{NaOH} = n_{CHO} \quad \text{(S1)}$$

$$OD \times \left(\frac{m_{OSA}}{M_{OSA}}\right) \times 2 = n_{CHO} \quad \text{(S2)}$$

where ΔV_{NaOH} is the consumed volume of NaOH solution (mL); c_{NaOH} the concentration of sodium hydroxide solution, which is 0.1 mol/L; m_{SA} the weight of OSA, which is 0.20 g; and M_{OSA} is the molecular weight of OSA units, which is 198.11 g/mol.

The substitution degree value was averaged from three independent samples.

Table S1. Degree of aldehyde substitution of OSA

**Samples**	m_{SA}**(g)**	c_{NaOH}**(mol/L)**	ΔV_{NaOH}**(ml)**	**Substitution Degree**	**Degree in Average**
1	0.20	0.1	17.02	84.30%	
2	0.20	0.1	17.56	86.97%	85.85%
3	0.20	0.1	17.42	86.28%	

Characterization of 3,3′-Dithiopropionic acid dihydrazide

3,3′-Dithiopropionic acid dihydrazide (DTP) was synthesized according to the literature[32]. The experimental values of ^{1}H NMR spectrum are in good agreement with the theoretical values. ^{1}H NMR (DMSO-d$_6$, 500 MHz, δ): 9.08 (s, 2H, NH_2-NH-CO), 4.21 (s, 4H, NH_2-NH-CO), 2.88 (t, 4H, CO-CH_2-CH_2), 2.40 (t, 4H, CO-CH_2-CH_2)

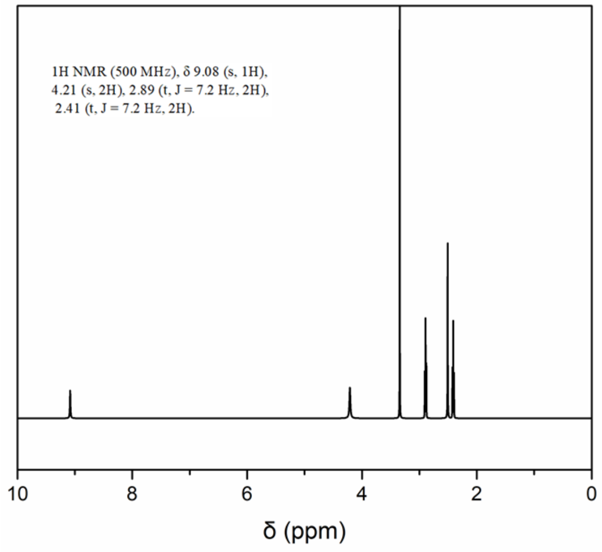

Figure S2. ^{1}H-NMR spectrum of DTP.

FT-IR Spectra of Ingredients and Hydrogels

The FT-IR spectra of OSA, CMC, OSA-DTP, CMC-OSA and CMC-OSA-DTP hydrogels were presented in Figure S3. The spectrum of OSA exhibits an absorption band at 1739 cm^{-1}, corresponding to the symmetric vibration peaks of C=O in aldehyde group, indicating the oxidation of alginate[45]. The peaks at 1614 cm^{-1} and 1412 cm^{-1} belong to the symmetric and antisymmetric stretching vibrations of $-COO^{-}$. In the spectra of CMC, the peak at 1419 cm^{-1} is due to the symmetrical deformation of $-CH_3$, and the band at 1128 cm^{-1} belongs to C-O stretching in glucosamine unit[39,46]. Compared with spectra of OSA, the FT-IR spectra of CMC-OSA-DTP hydrogel exhibits characteristic bands of $-COO^{-}$ at 1609 cm^{-1} and 1414 cm^{-1}, however, the characteristic peak of aldehyde is absent, corresponding to the cross-linking reactions in hydrogels[17]. In addition, the characteristic absorption of -C=N- appeared at around 1665 cm^{-1} is due to the imine bonds and acylhydrazone bonds. The peaks of imine bonds and acylhydrazone bonds are at similar wavenumbers, thus it is difficult to distinguish them. To prove the formation of different bonds, we prepared CMC-OSA hydrogel cross-linked by imine bonds and OSA-DTP cross-linked by imine bonds. A new peak of OSA-DTP hydrogel appears at 1664 cm^{-1} in the FT-IR spectra, verifying the formation of acylhydrazone bonds[34][35]. The spectra of CMC-OSA hydrogel exhibits the characteristic absorption peak at 1666 cm^{-1}, attributed to stretching vibration of imine bonds[36].

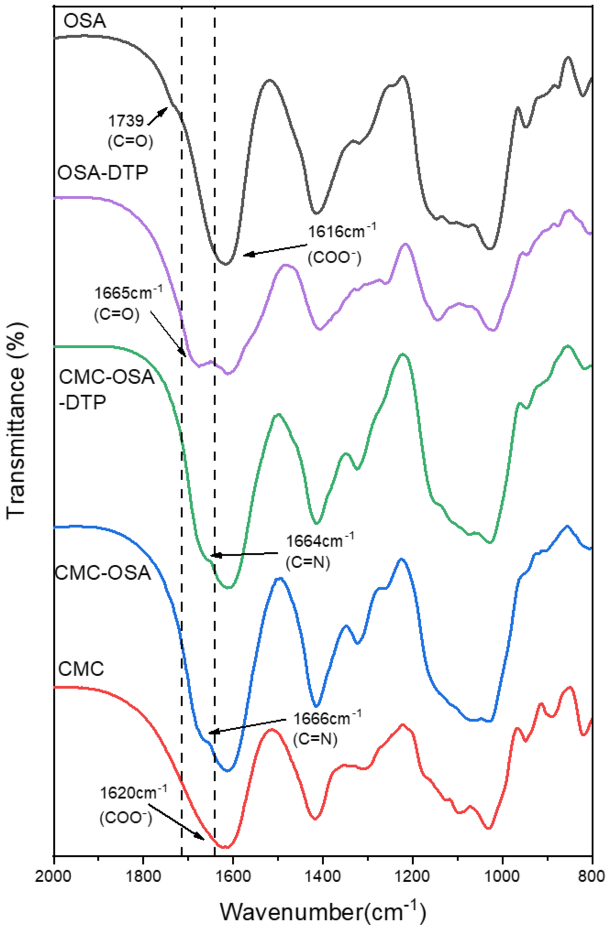

Figure S3. FT-IR spectra of oxidized sodium alginate (OSA), OSA-DTP hydrogel, CMC-OSA-DTP hydrogel, CMC-OSA hydrogel and carboxymethyl chitosan (CMC)

Swelling Property of CMC-OSA-DTP hydrogels

The swelling property experiment (Fig S4) performed with the CMC-OSA-DTP hydrogel which was dabbed away excess water carefully using a Kim wipe. The samples were immersed in PBS solution. In different time intervals, the swollen samples were taken out of the PBS solution. The samples were weighed after gently wiping out the excess water by Kim wipe. The weights were determined until no further change was detected. The swelling ratio was calculated according to the following equation:

$$\text{Swelling ratio} = (M_s - M_d)/M_d \times 100\% \quad \text{(S3)}$$

where M_d and M_s are the weights of hydrogels at the dry state and swollen state, respectively. As demonstrated in Figure S4, the swelling ratio of CMC-OSA-DTP hydrogel reached its equilibrium swelling ratio (1100%) in about 24 h. This high equilibrium swelling ratio is resulted from the presence of numerous hydrophilic groups in hydrogels, such as amino and carboxyl groups.

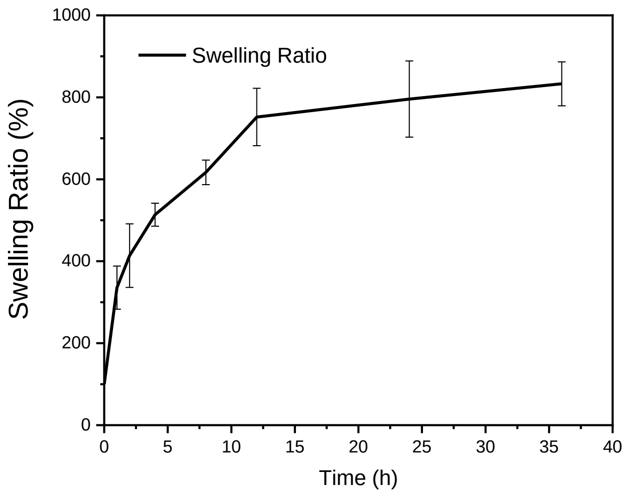

Figure S4. Swelling ratio of CMC-OSA-DTP hydrogels

UPLC spectra and results of sodium alginate and oxidized sodium alginate (OSA)

The sodium alginate and oxidized sodium alginate obtained after lyophilization were dissolved in deionized water and ultrasonically dispersed for 10 minutes to completely dissolve the two samples. Ultra high performance liquid chromatography (UPLC-SQD2, Waters, America) was used to determine the relative molecular mass of the two samples at room temperature. The dextran with a molecular weight of 670,000 was used as a reference, the injection speed was 0.5 mL/min, the injection volume was 3 μL, and the run time was 25 minutes. As shown in Figure S5, Mw of oxidized sodium alginate (OSA) is much smaller than Mw of sodium alginate which indicates that maybe sodium alginate is sensitive to oxidation and is degraded into smaller chains. In addition, the molecular weight distribution (Mw/Mn) of OSA is higher than sodium alginate, which further indicates the degradation of sodium alginate during the oxidation process. Hence, the oxidation process of sodium alginate should keep consistency to keep the reproducibility of hydrogel products.

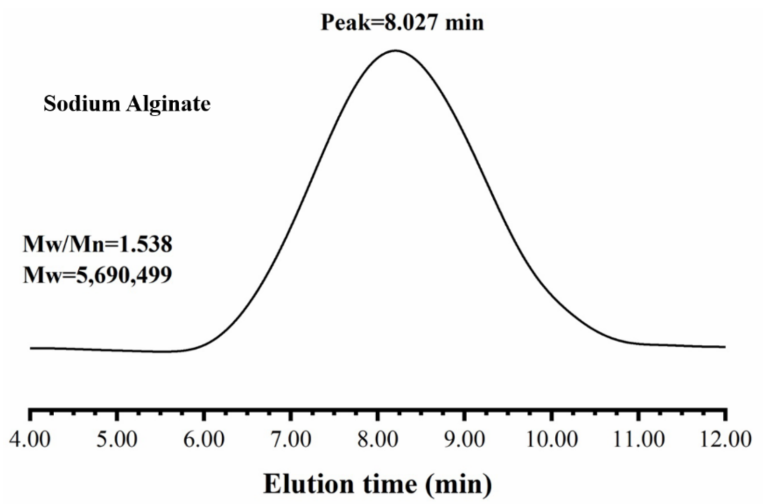

(a)

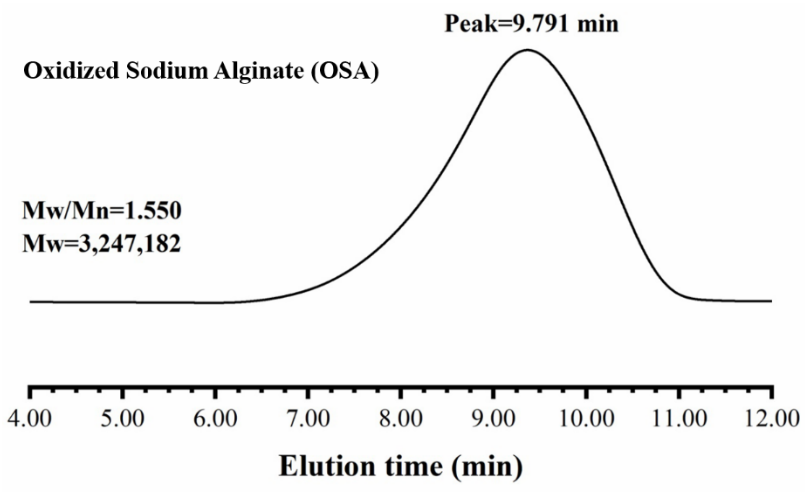

(b)

Figure S5. the molecular weight (Mw) and molecular weight distribution (Mw/Mn) of sodium alginate (a) and oxidized sodium alginate (b) by GPC

Note: All of references are listed in the maintext.
